# MicroRNA miR-34 Inhibits Human Pancreatic Cancer Tumor-Initiating Cells

**DOI:** 10.1371/journal.pone.0006816

**Published:** 2009-08-28

**Authors:** Qing Ji, Xinbao Hao, Min Zhang, Wenhua Tang, Meng Yang, Ling Li, Debing Xiang, Jeffrey T. DeSano, Guido T. Bommer, Daiming Fan, Eric R. Fearon, Theodore S. Lawrence, Liang Xu

**Affiliations:** 1 Department of Radiation Oncolog, University of Michigan, Ann Arbor, Michigan, United States of America; 2 Department of Internal Medicine, University of Michigan, Ann Arbor, Michigan, United States of America; 3 Department of Human Genetics, University of Michigan, Ann Arbor, Michigan, United States of America; 4 Department of Pathology, University of Michigan, Ann Arbor, Michigan, United States of America; 5 Comprehensive Cancer Center, University of Michigan, Ann Arbor, Michigan, United States of America; 6 State Key Laboratory of Cancer Biology and Institute of Digestive Diseases, Xijing Hospital, Fourth Military Medical University, Xi'an, Shaanxi, China; 7 Department of Hematology/Oncology, Hainan University Medical School, Haikou, Hainan, China; National Cancer Institute, United States of America

## Abstract

**Background:**

MicroRNAs (miRNAs) have been implicated in cancer initiation and progression via their ability to affect expression of genes and proteins that regulate cell proliferation and/or cell death. Transcription of the three miRNA miR-34 family members was recently found to be directly regulated by p53. Among the target proteins regulated by miR-34 are Notch pathway proteins and Bcl-2, suggesting the possibility of a role for miR-34 in the maintenance and survival of cancer stem cells.

**Methodology/Principal Findings:**

We examined the roles of miR-34 in p53-mutant human pancreatic cancer cell lines MiaPaCa2 and BxPC3, and the potential link to pancreatic cancer stem cells. Restoration of miR-34 expression in the pancreatic cancer cells by either transfection of miR-34 mimics or infection with lentiviral miR-34-MIF downregulated Bcl-2 and Notch1/2. miR-34 restoration significantly inhibited clonogenic cell growth and invasion, induced apoptosis and G1 and G2/M arrest in cell cycle, and sensitized the cells to chemotherapy and radiation. We identified that CD44+/CD133+ MiaPaCa2 cells are enriched with tumorsphere-forming and tumor-initiating cells or cancer stem/progenitor cells with high levels of Notch/Bcl-2 and loss of miR-34. More significantly, miR-34 restoration led to an 87% reduction of the tumor-initiating cell population, accompanied by significant inhibition of tumorsphere growth *in vitro* and tumor formation *in vivo*.

**Conclusions/Significance:**

Our results demonstrate that miR-34 may restore, at least in part, the tumor suppressing function of the p53 in p53-deficient human pancreatic cancer cells. Our data support the view that miR-34 may be involved in pancreatic cancer stem cell self-renewal, potentially via the direct modulation of downstream targets Bcl-2 and Notch, implying that miR-34 may play an important role in pancreatic cancer stem cell self-renewal and/or cell fate determination. Restoration of miR-34 may hold significant promise as a novel molecular therapy for human pancreatic cancer with loss of p53–miR34, potentially via inhibiting pancreatic cancer stem cells.

## Introduction

MicroRNAs (miRNAs) are a conserved class of non-coding 20–22 nt small RNAs that regulate gene and protein expression by binding to mRNA leading to mRNA degradation or inhibition of translation [1], [2]. miRNAs likely regulate diverse biological processes, including tissue differentiation and maintenance, and have contributing roles in varied disease processes, including cancer [2], [3], [4]. Emerging evidence suggests that miRNAs also play an essential role in stem cell self-renewal and differentiation by negatively regulating the expression of certain key genes involved in self-renewal and survival, so-called “stem cell genes” [1], [2], [4], [5]. Recently, the three members of the miR-34 family were found to be directly regulated by p53 and the functional activity of miR-34 indicated a potential role as a tumor suppressor [6], [7], [8], [9], [10], [11], [12]. We previously reported that the Bcl-2 protein is regulated directly by miR-34 [10]. The report from He et al. indicated that ectopic expression of miR-34 induces cell cycle arrest in both primary and tumor-derived cell lines, which is consistent with the ability of miR-34 to downregulate a program of genes promoting cell cycle progression [11]. We have recently shown that expression of miR-34s is dramatically reduced in p53-mutant gastric cancer cells and that the restoration of miR-34 expression inhibited the cancer cell growth [6]. Loss of miR-34 has been linked to chemoresistance of cancer [13]. miR-34a has been reported to be involved in p53-mediated apoptosis in colon cancer and pancreatic cancer [8], [9]. Chang et al. reported that 15 pancreatic cancer cell lines have at least a 2-fold reduction in miR-34a expression as compared to expression in normal pancreatic ductal epithelial cell lines [8]. Taken together, the published studies suggest miR-34 family members may have tumor suppressor function downstream of p53. In addition, because more than 50% of primary human cancers have mutations inactivating p53 function, the findings provided impetus to explore the functional restoration of miR-34 as a novel approach to inhibit cancers with p53 loss-of-function.

Another potential role for miR-34 in cancer initiation and progression may be a link to tumor-initiating cells or cancer stem cells (CSC). It has been reported that miR-34 targets Notch, c-Met and Bcl-2, genes involved in the self-renewal and survival of cancer stem cells [10], [11], [14]. At present, the linkages between p53, the downstream target miR-34 and presumptive pancreatic cancer stem cells are unknown. In the current study, we have examined the effects of functional restoration of miR-34 by miR-34 mimics and lentiviral miR-34a on human p53-mutant pancreatic cancer MiaPaCa2 cells, as well as the potential link to the pancreatic cancer stem cell self-renewal. Delineating the role of miR-34 in regulation of cell growth and tumor progression, and its potential link to tumor-initiating cells or cancer stem cells may provide a basis for exploring its potential as a novel treatment strategy.

## Materials and Methods

### Cell culture and reagents

Human pancreatic cancer cell lines and the normal human lung fibroblast cell line WI-38 were purchased from American Type Culture Collection and cultured in DMEM (HyClone, Logan, UT), supplemented with 10% fetal bovine serum (FBS; HyClone, Logan, UT). mi*RIDIAN* miRNA miR34a,b,c mimics and negative control miRNA mimic (NC mimic), mi*RIDIAN* miR-34 inhibitors and negative controls were obtained from Dharmacon (Chicago, IL) [6]. Bcl-2 3′UTR luciferase reporter plasmid or its mutant have been reported previously [10]. qRT-PCR primers and antibodies for Western blot are described in our recent publication [6].

### Transfection of miR-34 mimics

MiaPaCa2 cells were transfected 24 hr after being seeded in 6-well plates. miRNA mimics (100 pmol) in 200 µl of serum-free, antibiotic-free, medium were mixed with 5 µl of Lipofectamine 2000 transfection reagent (Invitrogen, Carlsbad, CA) dissolved in 200 µl of the same medium and allowed to stand at room temperature for 20 min. The resulting 400 µl transfection solutions were then added to each well containing 1.6 ml of medium. Six hr later, the cultures were replaced with 2 ml fresh medium supplemented with 10% FBS and antibiotics. For Western blot, cells were collected after an additional 48 hr.

### Lentiviral miR-34a infection

The feline immunodeficiency virus (FIV) lentiviral system expressing miR-34a (miR-34a-MIF) or vector control (MIF), as well as a lentiviral packaging system, were purchased from System Biosciences (SBI, Mountain View, CA). MiaPaCa2 and BxPC3 cells were infected with the FIV lentiviral system expressing miR-34a (miR-34a-MIF) or vector control (MIF), and the infected cells were selected via antibiotic resistance (Zeocin 50 µg/mL, Invitrogen), as we recently described[6].

### miR-34 Bcl-2-3′UTR reporter assay

MiaPaCa2 cells were transfected in 6-well plates with 2 µg of Bcl-2 3′UTR luciferase reporter plasmid or its mutant [6], [10], and 2 µg of the control β-galactosidase plasmid, per well, using Lipofectamine 2000 (Invitrogen). Cells were also co-transfected with 100 pmol of each miR-34 mimic or NC mimic, as indicated, using Lipofectamine 2000. Luciferase assays were performed 24 hr after transfection using Bright-Glo Luciferase Assay System (Promega). Luciferase activity was normalized relative to β-galactosidase activity detected by the β-galactosidase Assay System (Promega). In each case, mutant Bcl-2 3′UTR indicates the introduction of alterations into the seed complementary sites of Bcl-2 3′UTR [10].

### Colony formation and clonogenic assay

For colony formation assay, cells were transfected with miR-34 mimics or NC mimic for 24 hr, and then seeded in a 6-well plate in triplicate. 0.2 ml FBS was added per well on Day 5. After 9–10 days incubation, plates were gently washed with PBS and stained with 0.1% of crystal violet. Colonies with over 50 cells were manually counted. Plating efficiency was calculated by dividing the number of colonies formed in the treated group by that in control. For clonogenic survival assay, cells were transfected with miR-34 mimics or NC mimic for 24 hr, and then seeded in 6-well plates in triplicate at the desired cell density (200∼10,000 cells/well), followed by X-ray radiation. The cell survival curves were plotted using a linear-quadratic model and the radiation enhancement ratio (ER) was calculated as we previously described [15], [16], [17].

### Quantitative real-time RT-PCR (qRT-PCR)

qRT-PCR was performed to determine the expression levels of potential miR-34 target genes [6]. Twenty-four hr after miR-34 mimic transfection of MiaPaCa2 cells with miR-34 mimics (100 pmol per well in 6-well plates), the expression of potential target genes was measured by qRT-PCR with SYBR Green PCR System (TaqMan). Briefly, total RNA was extracted from the transfected cells using TRIZOL (Invitrogen) according to the manufacturer's instructions. Reverse transcription was performed by using a TaqMan Reverse Transcription Kit (Applied Biosystems). For qRT-PCR, 1 µl of gene primers with SYBR Green (Applied Biosystems) in 20 µl of reaction volume was applied. Primers were designed as: Bcl-2, forward, 5′-CAT GCT GGG GCC GTA CAG-3′, reverse, 5′-GAA CCG GCA CCT GCA CAC-3′; HMGA2, forward, 5′-TTT GTA ATC CCT TCA CAG TCC-3′, reverse, 5′-TTT CTC ACC CGC CCA C-3′; Notch1, forward, 5′-ATC CAG AGG CAA ACG GAG-3′, reverse, 5′-CAC ATG GCA ACA TCT AAC CC-3′; Notch2, forward, 5′-GGA CCC TGT CAT ACC CTC TT-3′, reverse, 5′-CAT GCT TAC GCT TTC GTT TT-3′; Notch3, forward, 5′-TGA TCG GCT CGG TAG TAA TG-3′, reverse, 5′-CAA CGC TCC CAG GTA GTC A-3′; Notch4, forward, 5′-TGC GAG GAA GAT ACG GAG TG-3′, reverse, 5′-CGG GAT CGG AAT GTT GG-3′; β-actin, forward, 5′-ATG CAG AAG GAG ATC ACT GC-3′, reverse, 5′-TCA TAG TCC GCC TAG AAG CA-3′. All reactions with TaqMan Universal PCR Master Mix (Applied Biosystems) were performed on the Mastercycler Realplex 2 (Eppendorf, Westbury, NY). Target gene mRNA levels were normalized to Actin mRNA according to the following formula: [2̂−(C_T_^target^ − C_T_^Actin^)] × 100%, where C_T_ is the threshold cycle. The relative expression was calculated by dividing the normalized target gene expression of the treated sample with that of the untreated control, with the value from NC mimic set as 1 arbitrary unit. For the target genes expression in the sorted cells, data were normalized to that of Actin (set Actin = 1000 arbitrary unit).

### Caspase-3 activation assay

Caspase activation in the transfected MiaPaCa2 cells was determined following the instructions of a Caspase-3 activation assay kit (BioVision, Mountain View, CA). Twenty-four hr after transfection, cells were lysed and the whole cell lysates (20 µg) were incubated with 25 µM fluorogenic substrate DEVD-AFC in a reaction buffer (containing 5 mM DTT) at 37°C for 2 hr. Proteolytic release of AFC was monitored at λex = 405 nm and λem = 500 nm using a fluorescence microplate reader (BMG LABTECH, Durham, NC). Relative caspase-3 activation was calculated by normalizing the fluorescence signal in each treated sample with that of NC mimic or MIF control set as 100 arbitrary unit [16].

### Cell cytotoxicity assay

MiaPaCa2 cells were transfected with miR-34 mimic or NC mimic for 24 hr, plated in 96-well plates (5,000 cells/well), and treated with serially diluted chemotherapeutic agents, in triplicates. After 96 h incubation, 20 µl/well CCK-8 reagent was added and incubated at 37°C for 1–3 hr. Optical density was measured at 450 nm and 650 nm using a microplate reader (BMG LABTECH, Durham, NC). IC_50_, the drug concentration that inhibits 50% cell growth was calculated by GraphPad Prism 5.0 (San Diego, CA) as we described previously [18].

### Cell cycle and apoptosis analysis

For cell cycle and apoptosis analysis by flow cytometry, MiaPaCa2 cells were transfected with miR-34 mimics or NC mimic in 6-well plates, trypsinized 24 hr later and washed with phosphate-buffered saline, and fixed in 70% ethanol on ice. After centrifugation, cells were stained with 50 µg/ml propidium iodide and 0.1 µg/ml RNase A, and analyzed by flow cytometry using a FACStar Plus™. Each histogram was constructed with the data from at least 5,000 events. Data were analyzed to calculate the percentage of cell population in each phase using the CellQuest software, as well as the % of cells in sub-G1 (Becton Dickinson) [18].

### CD44 and CD133 staining and cell sorting

MiaPaCa2 cells were incubated with PE-conjugated anti-human CD133/1 antibody and APC-conjugated anti-human CD44 antibody (Miltenyi Biotec, Aubum, CA, USA) in PBS containing 2% FBS. Isotype-matched mouse immunoglobulin served as controls. For flow cytometry, samples were analysed using a FACSCalibur flow cytometer and CellQuest software (BD Biosciences, San Jose, CA, USA). For cell sorting by flow cytometry, samples were analysed and sorted on a BD FACSVantage SE (BD Biosciences). Aliquots of CD133^+^ and CD133^−^ sorted cells were evaluated for purity with a FACSCalibur machine and CellQuest software (BD Biosciences), using PE-conjugated anti-human CD133/2 antibody (Miltenyi Biotec) [19].

### Tumorsphere culture

The sorted MiaPaCa2 cells were suspended in serum-free culture medium DMEM containing 1% N2 supplement, 2% B27 supplement, 1% antibotic-antimycotic (Invitrogen), 20 ng/ml human FGF-2 (Sigma), and 100 ng/ml EGF (Invitrogen), and plated in 24-well ultra-low attachment plates (Corning) 2,000 cells per well. 7–10 days later, plates were analyzed for tumorsphere formation and were quantified using an inverted microscope (Olympus) at 100X, 200X, and 400× magnifications. For subsequent quantification of cell numbers per tumorsphere, tumorspheres were collected with a 40 µm sieve (BD Biosciences, San Jose, CA) and disassociated with trypsin to make a single cell suspension. The viable cells were then counted using trypan blue exclusion.

### Animal model and in vivo tumor formation study

Five- to six-week old female athymic NCr-nu/nu nude mice were purchased from NCI. MiaPaCa2 cells were transfected with miR-34a mimic or NC mimic for 24 hr. Cells were collected and inoculated into nude mice subcutaneously (s.c.) on both flanks, after alcohol preparation of the skin, using a sterile 22-gauge needle with 0.2 ml cell suspension of 1×10^6^ cells, with manual restraint. The tumor sizes were measured using a caliper. Tumor volume was calculated using the formula: (length×width^2^)/2. On Day 38, all tumors were collected to measure the tumor weights. All animal experiments were done according to the protocol approved by University of Michigan Guidelines for Use and Care of Animals.

### Statistical analysis

Two-way ANOVA and two-tailed *t*-tests were employed to analyze the *in vitro* and *in vivo* data using Prism 5.0 software (GraphPad, San Diego, CA). *P*<0.05 was defined as statistically significant.

## Results

### Expression of miR-34s in human pancreatic cancer cell lines

**Figure 1:**
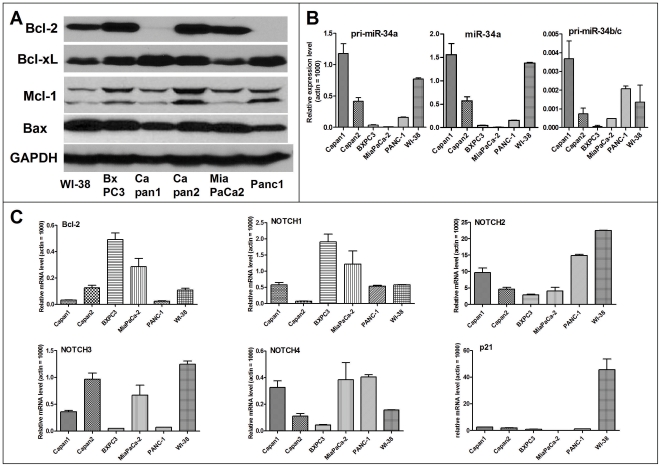
Expression of Bcl-2 family of proteins and miR-34s in human pancreatic cancer cell lines as well as normal human fibroblast cells WI-38. *A*, Western blot analysis. *B*, qRT-PCR analysis of relative expression levels of miR-34s. *C*, qRT-PCR analysis of the expression levels of miR-34 target genes in human pancreatic cancer cell lines as well as normal human fibroblast WI-38 cells. The cells were lyzed to extract total RNA for qRT-PCR, data were normalized to that of Actin and the relative levels are shown (Actin = 1000). Note p21 is a target gene of p53.

We examined a series of human pancreatic cancer cell lines, MiaPaCa2, BxPC3, Capan1, Capan2, Panc-1, and the normal human lung fibroblast cell line WI-38, for miR-34a,b,c expression. We also assessed in parallel the expression of presumptive miR-34-regulated target genes and proteins, using the primers and methods as we described recently [6]. MiaPaCa2 and BxPC3 cells have very low expression levels of both primary and mature miR-34a,b,c but high levels of the miR-34 target genes *BCL2* and *Notch1*, and different levels of *Notch2–4* (**Figure 1**). They also have low expression levels of p21 (**Figure 1** ***C***), another mRNA target of p53, consistent with the cell line's p53-mutant status. Based on these data and our observation that they are highly tumorigenic and reproducibly form tumorspheres *in vitro*, we chose the two cell lines for the current study.

### miR-34 restoration by transfection of MiaPaCa2 cells with miR-34 mimics

**Figure 2:**
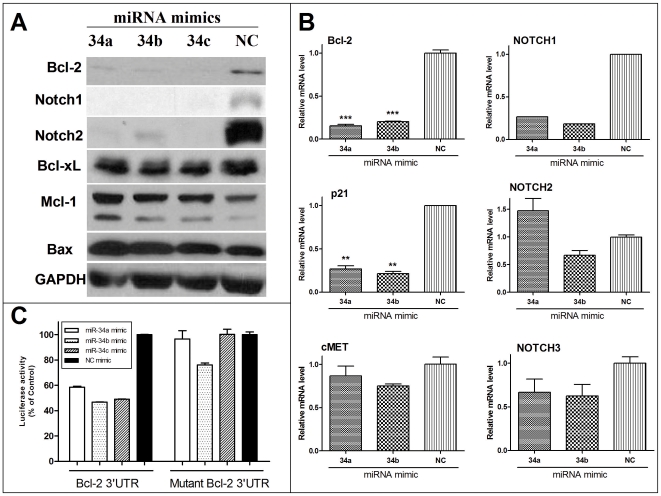
Restoration of miR-34 down-regulates target genes' expression. *A*, miR-34 restoration down-regulates target proteins Bcl-2, Notch1 and Notch2, no effects on Mcl-1. MiaPaCa2 cells were transfected with miR-34 mimics or non-specific control miRNA mimic (NC mimic) (100 pmol per well in 6-well plates by Lipofectamine 2000) for 48 hours, then collected for Western blot analysis. *B*, Quantitative real-time PCR analysis of the potential target genes' mRNA levels after miR-34 mimic transfection in MiaPaCa2 cells. **P<0.01, ***P<0.001, Student's *t*-test, n = 2. *C*, Bcl-2 3′UTR Luciferase Reporter Assay shows that the transfected miR-34 mimics are functional. MiaPaCa2 cells were co-transfected with the Bcl-2 3′UTR Luciferase Reporter or its mutant, b-gal vector, together with either miR-34 mimics or NC mimic. Luciferase assay was performed 24 hrs after transfection using Bright-Glo Luciferase Assay System. Luciferase activity was normalized relative to b-gal activity. Error bar indicates s.e.m.

To investigate the effects of miR-34 restoration on pancreatic cancer cells, we transfected the MiaPaCa2 cells with miR-34 mimics or non-specific control miRNA mimic (NC mimic). Western blot analysis (**Figure 2** ***A***) showed that transfection of miR-34 mimics downregulated expression of target genes, Bcl-2, Notch1 and Notch2 at the protein level, but had no effect on Bcl-xL and Mcl-1 expression, indicating the target gene knock-down by miR-34 mimics affects transcripts harbouring miR-34 target sites. miR-34a, miR-34b and miR-34c mimics all had similar activities. **Figure 2** ***B*** shows the qRT-PCR analysis of the potential target genes; miR-34 mimics potently inhibited *BCL2* and *Notch1* gene expression, consistent with the Western blot data. They also inhibited expression of p21 (**Figure 2** ***B***), another target of p53, but have limited effect on Notch3 and cMET. Interestingly, the Notch2 expression inhibition at the protein level by miR-34 was not accompanied by inhibition at the mRNA level, in agreement with previous reports that miRNA inhibits target gene expression post-transcriptionally, with or without mRNA degradation [11], [20].

To evaluate whether the transfected miR-34 mimics are functional, we carried out the Bcl-2 3′UTR reporter assay as we recently described [6], [10]. The transfected miR-34 mimics inhibited the luciferase reporter gene expression, which is controlled by Bcl-2 3′UTR in the promoter region (**Figure 2** ***C***). However, mutation in the Bcl-2 3′UTR complementary to the miR-34 seed sequence abolished this effect, indicating that the observed activity is sequence-specific. The results demonstrate that the transfected miR-34s are functional and confirm that Bcl-2 is a direct target of miR-34, consistent with earlier reports [8], [10], [21].

To evaluate the long-term effects of the miR-34 restoration, we also employed a lentiviral system to express miR-34a. The feline immunodeficiency virus lentiviral system expressing miR-34a (miR-34a-MIF), or vector control (MIF), was used to infect MiaPaCa2 and BxPC3 cells and the infected cell population was selected via Zeocin resistance [6]. **Figure S1** shows the characterization of expression changes in the miR-34a-MIF cells. Western blot analysis revealed that Bcl-2 protein was down-regulated in miR-34a-MIF cells as compared with the MIF vector control cells (**Figure S1** ***A***), consistent with qRT-PCR analysis of the Bcl-2 mRNA level (**Figure S1** ***B***). Bcl-2 3′UTR Luciferase Reporter Assay showed that the miR-34a is functional in the miR-34a-MIF cells (**Figure S1** ***C***).

### miR-34 restoration inhibits MiaPaCa2 cell clonogenic growth and leads to caspase-3 activation and apoptosis

**Figure 3:**
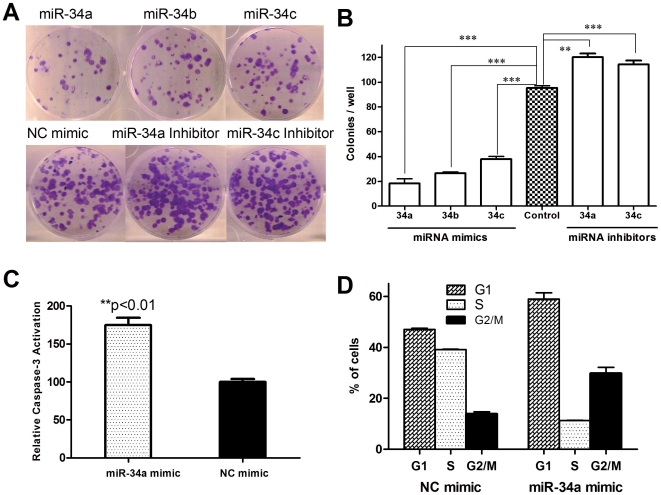
Restoration of miR-34 inhibits the clonogenic growth of MiaPaCa2 cells, whereas inhibition of miR-34 promotes cell growth. MiaPaCa2 cells were transfected with miR-34 mimics or inhibitors, 24 hr later the cells were seeded in 6-well plates (200 cells/well, in triplicates). After 12–14 days incubation, the plates were gently washed with PBS and stained with 0.1% crystal violet. *A*, representative pictures of the colonies. *B*, Colonies with over 50 cells were counted. *C*, Restoration of miR-34 leads to caspase-3 activation. Caspase-3 activation assay was carried out as described in in *Materials and Methods*. Fold increase of fluorescence signal was calculated by dividing the normalized signal in each treated sample with that in the untreated control. **P<0.01, ***P<0.001, Student's *t*-test, n = 3. *D*, Cell cycle distribution of MiaPaCa2 cells transfected with miR-34 mimics. Cell cycle analysis was performed 1 day after transfection. Cells were stained with propidium iodide after ethanol fixation and analyzed by flow cytometry.

After validating that the transfected miR-34 mimics were functional, we carried out a clonogenic assay to examine the effects of miR-34 restoration on cell growth. As shown in **Figure 3** ***A–B***, miR-34 restoration significantly inhibited the clonogenic cell growth, with miR-34a mimic inducing >80% inhibition of colony formation compared to NC mimic (18.3±3.8 colonies/well vs. 95.3±1.8% colonies/well). Similar results were observed in the miR-34a-MIF cells which grew slower than MIF control cells, as indicated by both the significantly reduced number of Trypan Blue-excluding viable cells in the cell growth assay (**Figure S1** ***D***) and the reduced colony formation (**Figure S1** ***E***). We also examined the effect of inhibition of endogenous miR-34 on cell growth by mi*RIDIAN* miR-34 inhibitors. They are single-stranded chemically enhanced oligonucleotides that can effectively inhibit the endogenous mature miR-34. miR-34 inhibitors induced an almost 20% increase in clonogenic growth as compared with control (120.3±2.9 colonies/well vs. 95.3±1.8 colonies/well) (**Figure 3** ***A–B***). We also carried out a cell invasion assay in MiaPaCa2 cells with miR-34 restoration by both miR-34a mimic and miR-34a-MIF. miR-34 significantly inhibited the invasion potential of MiaPaCa2 cells (**Figure S2**). Our results demonstrate that miR-34 is involved in MiaPaCa2 cell growth; miR-34 restoration inhibits the clonogenic growth, and inhibition of endogenous miR-34 by miR-34 inhibitors promotes the growth. Our data are consistent with the reported tumor suppressor function of miR-34 [6], [8], [9], [11], [22].

Since p53 tumor suppressor function is mediated in part via induction of apoptosis [23], [24], we examined the effect of miR-34 restoration on apoptosis-induction in MiaPaCa2 cells transfected with miR-34 mimics. As shown in **Figure 3** ***C***, transient transfection of miR-34 mimics resulted in significantly increased activation of caspase-3, a key indication of the cells undergoing apoptosis [25]. We also evaluated the effect of miR-34 mimics on cell cycle. miR-34 mimics resulted in significant G1 and G2/M arrest and a reduction of cells in S phase (**Figure 3** ***D***), consistent with other reports on miR-34 restoration in various cancer models [6], [7], [8], [10], [11], [21], [22], [26]. This effect on cell cycle is similar to that of p53 restoration as we previously reported [23], [24], [27], [28], indicating that miR-34 restoration can exert effects akin to restoration of p53 tumor suppressor function, at least in part, in the cells with p53 loss of function.

### miR-34 restoration sensitizes MiaPaCa2 cells to chemo- and radiotherapy

**Figure 4:**
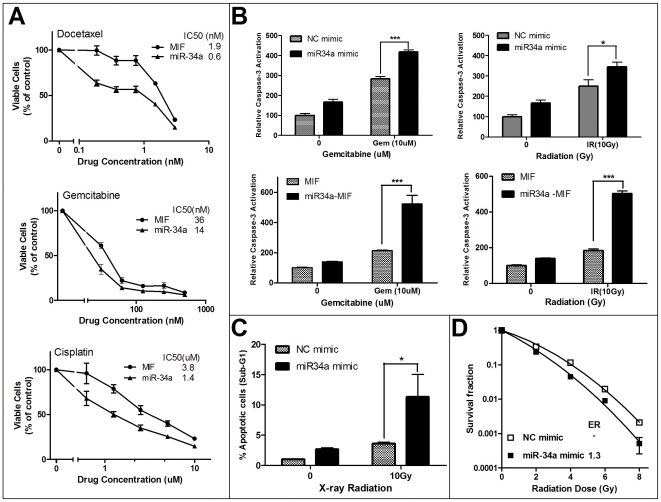
Restoration of miR-34 sensitizes MiaPaCa2 cells to chemotherapy and radiation. *A*, miR-34 restoration sensitizes the cells to chemotherapeutic agents. The MTT-based cytotoxicity assay was carried out using the Zeocin-resistant stable MiaPaCa2-miR-34a-MIF and MiaPaCa2-MIF cells. *B*, miR-34 restoration increases caspase-3 activation induced by gemcitabine or X-ray radiation in MiaPaCa2 cells. Relative caspase-3 activation was calculated by normalizing the fluorescence signal in each treated sample with that of the NC mimic or MIF control as 100. *P<0.05, ***P<0.001, Student's *t*-test, n = 3. *C*, miR-34 restoration increases radiation-induced apoptosis in MiaPaCa-2 cells. Cells were transfected with miR-34a mimic or NC mimic. 24 hr later, the cells were subjected to X-ray radiation. The cells were collected after another 48 hr, stained with propidium iodide after ethanol fixation, and analyzed by flow cytometry for the % of cells in sub-G1 phase. *P<0.05, Student's t-test, n = 2. *D*, miR-34 restoration radiosensitized MiaPaCa-2 cells. The clonogenic assay was carried out as described in *Materials and Methods* Data are shown as mean +/− SD (n = 3).

Next, we examined whether miR-34 restoration could sensitize the pancreatic cancer cells with a high level of endogenous Bcl-2 expression to chemo- and radiotherapy. The WST-1-based cytotoxicity assay was used as we recently described [6], [18] to evaluate the cells' response to three chemotherapeutic agents, docetaxel, cisplatin and gemcitabine, all of which are currently used for pancreatic cancer chemotherapy. As shown in **Figure 4** ***A***, miR-34 restoration in MiaPaCa2 cells rendered the cells 2–3-fold more sensitive to the chemotherapeutic agents, as compared with the vector control cells, based on IC50 data. In apoptosis assays, miR-34a restoration significantly increased Gemcitabine or radiation induced caspase-3 activation (**Figure 4** ***B***) and sub-G1 cells (**Figure 4** ***C***). We have also carried out clonogenic assay, miR-34a mimic sensitized MiaPaCa2 cells to X-ray radiation, with a radiation enhancement ratio (ER) = 1.3 (**Figure 4** ***D***). Our data demonstrate that miR-34 restoration can overcome chemo-/radioresistance of the pancreatic cancer cells that have high levels of Bcl-2 and low basal levels of miR-34s, and are dependent on Bcl-2 for survival and resistance to therapy.

### CD44+/CD133+ cells are tumorsphere-forming and tumor-initiating cells with high Bcl-2 and loss of miR-34 expression

**Figure 5:**
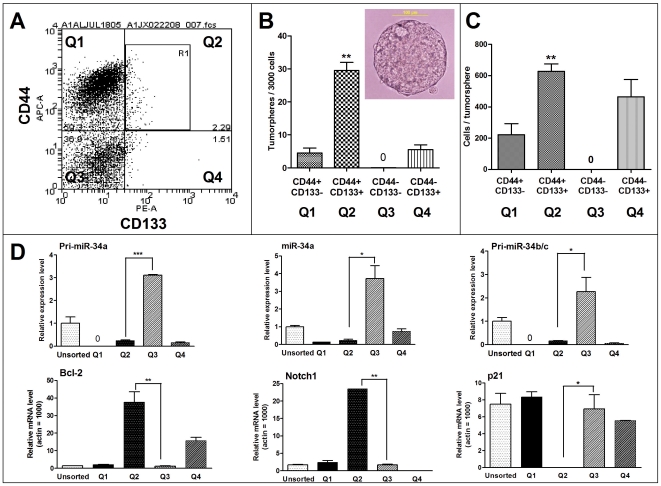
MiaPaCa2 CD44+/CD133+ cells are tumorsphere-forming cells that have high Bcl-2 and loss of miR-34. *A*, CD44 and CD133 staining of MiaPaCa2 cells. MiaPaCa2 cells were stained by anti-CD44-APC and anti-CD133-PE and sorted by FACS. Around 1–2% cells are CD44+/CD133+ double-positive (Q2). *B–C*, Tumorsphere culture of the sorted cells. The sorted cells were plated for tumorsphere culture as described in *Materials and Methods*. 7–10 days later, tumorspheres were counted (*B*). The insert shows a representative tumorsphere from CD44+/CD133+ MiaPaCa2 cells. *C*. Quantification of cell numbers per tumorsphere. Tumorspheres were collected with 40 um filter and dissociated with trypsin to make a single cell suspension. Cells were counted with trypan blue exclusion and data are presented as number of cells per tumorsphere. CD44+/CD133+ cells are tumorsphere-forming cells whereas CD44−/CD133− cells did not grow tumorspheres. *P<0.05, **P<0.01, ***P<0.001, Student's *t*-test, n = 3. *D*, qRT-PCR analysis of the expression levels of miR-34 and target genes in the sorted MiaPaCa2 cells. The sorted cells were lyzed to extract total RNA for qRT-PCR. The miR-34 expression data were normalized to that of Actin and the relative levels are shown (set unsorted cells = 1). For the target genes expression, data were normalized to that of Actin (set Actin = 1000). CD44+/CD133+ (Q2) cells have high levels of Bcl-2 and Notch1 but loss of miR-34a/b/c as compared with CD44−/CD133− (Q3) cells. *P<0.05, **P<0.01, ***P<0.001, Student's *t*-test, n = 2.

To investigate the potential effect of miR-34 restoration on tumor-initiating cells in the MiaPaCa2 cell line, we first examined the tumor-initiating cell or cancer stem cell population in MiaPaCa2 cells with various cell surface markers. Both CD44 [29] and CD133 [19], [30] have been used as markers to identify the pancreatic cancer stem cells from human tumor tissues. However, there is no report on the cancer stem cells in MiaPaCa2 cells. We evaluated the CD44 and CD133 status in MiaPaCa2 cells by immunofluorescent staining and FACS sorting. About 60% cells are CD44+ and 3–5% cells are CD133+, however, only 1–2% cells are CD44+/CD133+ double-positive (Q2 in **Figure 5** ***A***). To examine the self-renewal potential of the cells with different surface marker profiles, we undertook the tumorsphere culture of the sorted cells in a special ultra-low attachment culture plate with conditional medium for tumorsphere culture [29]. Seven to ten days later, the CD44+/CD133+ double-positive MiaPaCa2 cells grew typical tumorspheres but not the CD44−/CD133− double-negative cells, whereas CD44+/CD133− or CD44−/CD133+ single-positive cells had fewer and smaller spheres (**Figure 5** ***B–C***). A representative tumorsphere from CD44+/CD133+ cells is shown in **Figure 5** ***B*** (the insert). We also carried out a limiting dilution tumor-initiation assay in nude mice using the sorted cells, and data are summarized in **Table 1**. CD44−/CD133− cells did not form tumor, whereas 1×10^4^ CD44+/CD133+ cells formed tumor in 4 out of 4 mice, 1×10^3^ CD44+/CD133+ cells formed tumor in 2 out of 4 mice, and no tumor formed with 1×10^2^ cells. Thus, our data demonstrate that the CD44+/CD133+ double-positive MiaPaCa2 cells are enriched with tumor-initiating cells or cancer stem/progenitor cells capable of self-renewal, but the CD44−/CD133− double-negative population contains no such cells. Our results also suggest that CD44/CD133 are suitable markers for tumor-initiating cells in the MiaPaCa2 cell line.

**Table 1:** Tumor-initiation study.

Cells inoculated	1×10^4^	1×10^3^	1×10^2^
CD44+/133+	4/4**	2/4	0/4
CD44−/133−	0/4	0/4	0/4

MiaPaCa2 cells were stained and sorted for CD44+/CD133+ and CD44−/CD133− cells, and then s.q. inoculated in nude mice. Data are number of mice with tumors formed/number of mice inoculated.

** *P*<0.01, *Chi*-square test.

Next, we carried out qRT-PCR analysis of the sorted MiaPaCa2 cells to assess whether there is any difference in these populations as to the expression levels of miR-34 and its target genes. As shown in **Figure 5** ***D***, the CD44+/CD133+ (Q2) cells have a high level of Bcl-2 expression but loss of miR-34a/b/c as compared with CD44−/CD133− (Q3) cells or the unsorted (total) cells. There is an inverse correlation in the expression levels of miR-34 and Bcl-2 in Q2 versus Q3, e.g., Q2 cells (with enriched cancer stem cells) have high Bcl-2 and low miR-34, Q3 cells (non-tumorigenic cells) have low Bcl-2 and high miR-34 levels. Our results are consistent with the notion that Bcl-2 is a direct target of miR-34 and miR-34 potently inhibits Bcl-2 expression. More importantly, our results demonstrate for the first time that the CD44+/CD133+ tumor-initiating cells, or pancreatic cancer stem cells, have a low level of miR-34 accompanied by a high level of Bcl-2, suggesting a potential link of miR-34 and its target Bcl-2 to pancreatic cancer stem cells.

### miR-34 restoration leads to a significant reduction of CD44+/CD133+ cells and inhibition of tumorsphere growth

**Figure 6:**
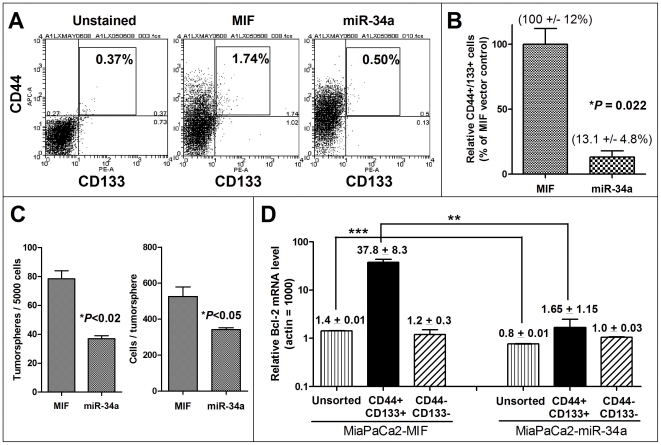
Restoration of miR-34 by MIF lentiviral system decreases the CD44+/CD133+ MiaPaCa2 cells and inhibits tumorspheres from the sorted CD44+/CD133+ cells. *A–B*, CD44 and CD133 FACS analysis of the MiaPaCa2-miR-34a-MIF and MiaPaCa2-MIF cells. miR-34a restoration significantly reduced the CD44+/CD133+ cells. Values are mean±s.e.m, n = 2. *C*, miR-34a restoration inhibits tumorspheres from the sorted CD44+/CD133+ cells. The cell sorting and tumorsphere culture were as described in *Materials and Methods*. 7–10 days later, tumorspheres were counted and the cell numbers per tumorsphere were quantified. Tumorspheres were collected with 40 um filter (BD) and dissociated with trypsin to make a single cell suspension. Cells were counted with trypan blue exclusion and data are presented as number of cells per tumorsphere. *P<0.05, Student's *t*-test, n = 3. *D*, qRT-PCR analysis of Bcl-2 mRNA levels in the sorted cells with or without miR-34a restoration. Data are shown as relative mRNA levels normalized to that of Actin = 1000 arbitrary units. Values are mean±s.d, n = 2. miR-34a restoration led to almost 23-fold reduction of Bcl-2 mRNA in CD44+/CD133+ cells, compared with 43% reduction in total population. **P<0.01, ***P<0.001, one-way ANOVA and Student's *t*-test, n = 2.

**Figure 7:**
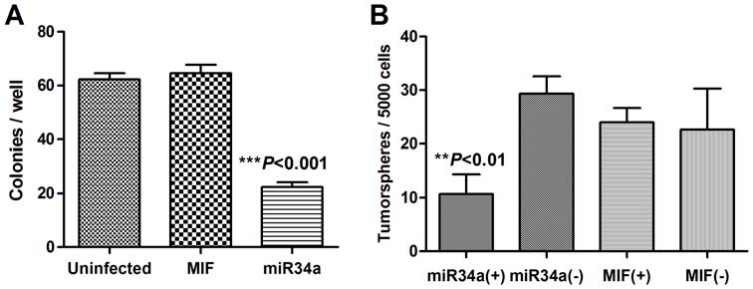
Restoration of miR34a inhibits the clonogenic growth and tumorspheres of BxPC-3 cells. BxPC-3 cells were infected with lentiviral miR-34a expression system (miR-34a) or vector control (MIF), and the infected cells were sorted for GFP positive cells by FACS. The sorted cells were plated for either colony formation (*A*) or tumorsphere culture (*B*) as described in *Materials and Methods*. **P<0.01, ***P<0.001, Student's *t*-test, n = 3.

Our above results have shown that miR-34 potently inhibits Bcl-2 expression and cell growth and increases cell death and response to chemo-/radiotherapy in the overall population of MiaPaCa-2 cells. We have also shown that the CD44+/CD133+ tumorsphere-forming and tumor-initiating cells have high Bcl-2 and loss of miR-34 expression, indicating that miR-34 and its target Bcl-2 might be involved in cancer stem cells. To investigate the potential role of miR-34 in pancreatic cancer stem cells, we examined whether miR-34 restoration could inhibit the CD44+/CD133+ cells and their self-renewal potential. **Figure 6** ***A*** shows that miR-34a restoration significantly decreased the CD44+/CD133+ cells (0.58±0.08% versus MIF control 1.93±0.19%, *P* = 0.022, n = 2), an 87% reduction (**Figure 6** ***B***). This miR-34a-induced reduction of the CD44+/CD133+ cells was accompanied by reduced tumorsphere formation and smaller size of the tumorspheres (**Figure 6** ***C***), and associated with a significant reduction of Bcl-2 expression in the CD44+/CD133+ tumorsphere-forming cells (**Figure 6** ***D***). Interestingly, although miR-34a inhibited the Bcl-2 expression in the total population as well as in both the sorted CD44+/CD133+ cells and CD44−/CD133− cells, the effect of miR-34a on the CD44+/CD133+ population was most dramatic (miR-34a 1.65±1.15 vs. MIF 37.8±8.3, almost a 23-fold reduction at Bcl-2 mRNA level, compared with that of the total populations: miR-34a 0.8±0.01 vs. MIF 1.4±0.03, a 43% reduction) (**Figure 6** ***D***). Similar results on tumorspheres were observed with the total cells from the miR-34a-MIF cells (**Figure S3**). Our data provide the first evidence that miR-34 is able to inhibit CD44+/CD133+ tumorsphere-forming and tumor-initiating cancer stem cells in p53-mutant pancreatic cancer, implying that miR-34 might play a role in the self-renewal of pancreatic cancer stem cells. Similar results were also observed in BxPC3 cells, where lentiviral miR-34a restoration significantly inhibited the clonogenic growth (**Figure 7** ***A***) and tumorspheres (**Figure 7** ***B***).

### miR-34 restoration inhibits the MiaPaCa2 tumor formation in nude mice

**Figure 8:**
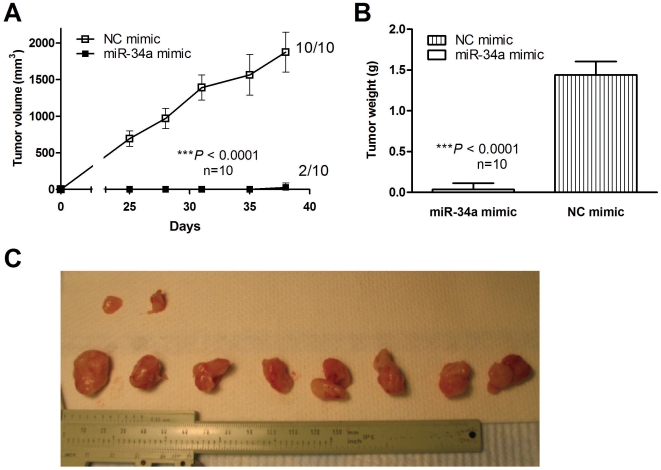
miR-34 restoration inhibits the MiaPaCa2 tumor initiation in nude mice. MiaPaCa2 cells were transfected with miR-34a mimic or NC mimic for 24 hours. Cells were collected and inoculated into female athymic nude mice subcutaneously (s.c.) on both sides of flank, 1×10^6^ cells/0.2 ml. The tumor sizes were measured using a caliper (*A*). Tumor volume was calculated using the formula: (length×width^2^)/2. On Day 38, all tumors were collected to measure the tumor weights (*B*). *C*, Picture of the tumors. ***P<0.0001, two-way ANOVA, n = 10.

We carried out studies to examine the effect of miR-34 restoration on tumor initiation *in vivo*. MiaPaCa2 cells were transfected with miR-34a mimic or NC mimic for 24 hours. Cells were collected and inoculated into female athymic nude mice subcutaneously (s.c.). As shown in **Figure 8** ***A***, miR-34a restoration significantly inhibited MiaPaCa2 tumor formation *in vivo* (miR-34a mimic 2/10 versus NC mimic 10/10, *P*<0.0001, n = 10). The average tumor sizes were also significantly smaller than that with control miRNA (**Figure 8** ***B–C***).

## Discussion

Our results show that miR-34 restoration in human pancreatic cancer MiaPaCa2 and BxPC3 cells inhibited the expression of target genes, Bcl-2, Notch1 and Notch2; significantly inhibited clonogenic cell growth and invasion; induced apoptosis and G1 and G2/M arrest; and sensitized the cells to chemotherapy and radiation. Our study demonstrates that miR-34 may restore, at least in part, the tumor suppressing function of p53 in p53-deficient cancer cells. We have also identified that CD44+/CD133+ MiaPaCa2 cells are enriched with tumorsphere-forming and tumor-initiating cells or CSC with high levels of Notch/Bcl-2 and loss of miR-34s. More significantly, we show that miR-34 restoration led to an 87% reduction of the CD44+/CD133+ CSC, accompanied by significant inhibition of tumorsphere growth *in vitro* as well as tumor formation *in vivo*. Our data support the view that miR-34 may be involved in pancreatic cancer stem cell self-renewal, potentially via the direct modulation of downstream targets Bcl-2 and Notch, implying that miR-34 may play an important role in pancreatic cancer stem cell self-renewal and/or cell fate determination, at least in the p53-mutant MiaPaCa2 model. These findings suggest that miR-34 mimics may hold significant promise as a novel molecular therapy for human pancreatic cancer with loss of p53–miR34, potentially via modulating pancreatic cancer stem cells.

A potential advantage of miRNAs as therapeutics is that miRNA can modulate multiple cellular pathways simultaneously [6]. One major difference between miRNA and siRNA is that siRNA is specific to a particular target via perfect sequence match, while one miRNA can bind to multiple 3′UTRs of target mRNAs via 6–8 nucleotides root-sequence match and thus inhibits multiple target genes. Our recent study with multidrug resistant cancer cells found that Bcl-2 upregulation and p53 downregulation are involved in chemoresistance [31]. Thus, inhibition of Bcl-2 function and restoration of p53 simultaneously represents a promising strategy to overcome drug resistance and improve efficacy for the treatment of p53-mutant pancreatic cancer. This strategy was explored in the current study, where p53 downstream target miR-34 was restored in p53-mutant pancreatic cancer MiaPaCa2 cells with a high level of Bcl-2 and low levels of miR-34s, resulting in downregulation of Bcl-2 and Notch1-2, together with the inhibited clonogenic cell growth and invasion; increased apoptosis and G1 and G2/M arrest in cell cycle; and sensitization to chemotherapy and radiation. miR-34 restoration could thus re-build, at least in part, the p53 tumor suppressing signalling network in pancreatic cancer cells lacking functional p53. This multi-mode action of miR-34 provides a therapeutic advantage over the siRNA-based therapies in that miR-34 has multiple targets, can work on multiple cell signalling pathways at the same time, leading to synergistic effects which may translate into improved clinical efficacy for pancreatic cancer patients with p53 deficiency and advanced disease.

Another important finding from the current study is that our data provide a potential link between the tumor suppressor miR-34 and the tumor-initiating cells or cancer stem cells. CSCs are a small subpopulation of cells capable of self-renewal and differentiation and have been identified in a variety of tumors [29], [32], [33], [34], [35]. CSC may be responsible for tumor initiation, progression, metastasis and resistance to therapy [36], [37], [38], [39]. If this is the case, it would suggest that cancer therapy should be directed against both the resting CSC and the proliferating cancer cells [37]. This may be possible if specific stem cell signals are inhibited using molecular therapy, while at the same time attacking proliferating cells by conventional therapies [33], [38]. Stem cells are defined by their ability to undergo self-renewal, as well as multi-lineage differentiation [40]. Tumorsphere culture has been widely used to assess the self-renewal potential of stem cells and cancer stem cells [5], [14], [32], [39]. Our results show that miR-34 restoration caused an 87% reduction of the CD44+/CD133+ tumorsphere-forming and tumor-initiating CSC in MiaPaCa2 cells with p53 loss of function, accompanied by a significant inhibition of tumorsphere growth *in vitro* and tumor formation *in vivo*. The miR-34-mediated reduction of the CD44+/CD133+ CSC is associated with the potent and simultaneous inhibition of its downstream target genes Notch and Bcl-2, genes involved in stem cells self-renewal and survival, so-called “stem cell genes” or “stemness genes” [6], [41], [42], [43]. Here again, miR-34 shows the advantage of its multi-target potential, as both stem cell genes Notch and Bcl-2 are inhibited by miR-34 at the same time, a potent synergy may be achieved in blocking both Notch signalling pathway and the anti-apoptotic function of Bcl-2 in tumor-initiating cells or cancer stem cells. Notch signalling pathway has been implicated in cancer stem cells [6], [44] and has strong potential as a promising target for pancreatic cancer [45]. It has been reported that Notch signaling regulates stem cell numbers *in vitro* and *in vivo* [46]. In a brain tumor model, inhibiting the Notch pathway indeed depleted CD133+ brain cancer stem cells and blocked tumor initiation [44], consistent with our findings in pancreatic cancer model. However, our study is the first report showing that miRNA miR-34 inhibits pancreatic CD44+/CD133+ CSC, potentially via inhibiting downstream target “stem cell genes” such as Notch and Bcl-2. Interestingly, miR-34a and miR-34b are among the short-list of the stem cell-specific miRNAs discovered by Dr. Sharp's group in their pioneer miRNA study [47], supporting the link of miR-34 to CSC. We are currently carrying out more detailed mechanism studies to delineate the involvement of Notch signaling pathway in miR-34-induced inhibition of pancreatic CSC and its role in chemo/radiosensitization of pancreatic cancer with p53 loss of function.

According to the CSC model [33], [48] that gains increasing attention recently, for a cancer therapy to be effective and curative, these highly resistant tumor-initiating cells must be eliminated. Currently, much research is aimed at identifying the genetic, epigenetic and protein signatures unique for cancer stem cells and distinguishing them from non-tumorigenic cells. Our current study delineates some of those distinguishing characteristics. Our results show that the CD44+/CD133+ MiaPaCa2 cells, even though only comprising 1–2% of total cell population, have much higher levels of Bcl-2 and Notch1, and lower levels of miR-34a,b,c, while CD44−/CD133− cells have levels comparable to that of total population. miR-34a restoration resulted in an 87% reduction of the CD44+/CD133+ cells, accompanied by significant inhibition of tumorsphere growth *in vitro* as well as tumor formation *in vivo*. More interestingly, this effect was associated with a 23-fold downregulation of Bcl-2 in the CD44+/CD133+ cells, while only 43% downregulation in total cells. These data indicate that the CD44+/CD133+ cells were the target cells of miR-34, i.e., miR-34 exerts its tumor-suppressing activity via inhibiting the CD44+/CD133+ cells. Our results also imply that current gene expression profiling studies with the total cell population may not reflect the real genetic signature of the small side-population of CSC which only comprises 1–2% of total cells. As demonstrated in our study, one has to isolate the specific CSC population in order to identify its gene expression signature and to address whether a molecular therapy is indeed hitting its target(s) in CSC, which has significant implication in our efforts to discover and develop novel therapies targeting CSC. Currently, we are using gene and miRNA arrays with the sorted tumor cells for the genetic and epigenetic profiling of cancer stem cells.

In conclusion, our study demonstrates that miR-34 may restore, at least in part, the tumor suppressing function of p53 in p53-deficient human pancreatic cancer cells. More significantly, miR-34 restoration inhibits the CD44+/CD133+ tumor-initiating cells or CSC, accompanied by significant inhibition of tumorsphere growth *in vitro* and tumor formation *in vivo*. Our data provide the first evidence that miR-34 is involved in pancreatic CSC self-renewal, potentially via the direct modulation of downstream targets Notch and Bcl-2. Our results provide novel insight into how miR-34 works in pancreatic cancer cells with p53 loss of function. By modulating CSC, the restoration of tumor suppressor miR-34 may provide a novel therapeutic approach for p53-deficient pancreatic cancer.

## Supporting Information

Figure S1 Characterization of the Zeocin-resistant stable MiaPaCa2-miR-34a cells. MiaPaCa2 cells were infected with feline immunodeficiency virus (FIV) lentiviral system expressing miR-34a (miR-34a-MIF) or control (MIF), and selected for stable cells by Zeocin-resistance. A, Western blot shows Bcl-2 protein is downregulated in miR-34a-MIF clone. B, qRT-PCR analysis shows that the target gene Bcl-2 is downregulated in miR-34-MIF clone. C, Bcl-2 3′UTR Luciferase Reporter Assay shows that the miR-34a is functional in miR-34-MIF clone. Error bar indicates S.D. D, miR-34a-MIF cells grows slower than MIF control cells. Cells were plated in a 24-well plate, at every 24 h, cells were harvested in triplicate wells and the viable cells were counted by Trypan Blue exclusion. E, Colony formation assay shows the miR-34a inhibits clonogenic growth of the miR-34-MIF. Cells were seeded in 6-well plate (200 cells/well) in triplicates. After 12–14 days incubation, plates were stained with 0.1% crystal violet. Colonies with over 50 cells were counted. **P<0.01, ***P<0.001, Student's t-test, n = 3.
(1.02 MB TIF)

Figure S2 Restoration of miR-34 inhibits the invasion of MiaPaCa2 cells. Cell invasion assay was carried out using Transwells Invasion Kit (Corning Costar) in 24-well tissue culture plates. A–B. MiaPaCa2 cells were transfected with miR-34a mimic or NC mimic and placed in the Transwell inserts, cultured for two days, observed under microscope (A) and quantified (B). C–D. The stable MiaPaCa2-miR-34a-MIF or MiaPaCa2-MIF cells were placed in the Transwell inserts, cultured for two days, observed under microscope (C) and quantified (D).
(7.60 MB TIF)

Figure S3 Restoration of miR-34 by MIF lentiviral system inhibited MiaPaCa2 tumorspheres. The stable MiaPaCa2-miR-34a-MIF or MiaPaCa2-MIF cells were plated for tumorsphere formation as described in Materials and Methods. 7–10 days later, tumorspheres were observed under microscope (A) and quantified (B). C. Quantification of cell numbers per tumorsphere. Tumorspheres were collected with a 40 um filter (BD), and dissociated with trypsin for single cell suspension. Cells were counted with trypan blue exclusion and data are presented as number of cells per tumorsphere. **P<0.01, ***P<0.001, Student's t-test, n = 3.
(3.07 MB TIF)
