# Supplementary figures and images for: MicroRNA miR-34 Inhibits Human Pancreatic Cancer Tumor-Initiating Cells
Source: PLoS One. 2009 Aug 28;4(8):e6816. doi: 10.1371/journal.pone.0006816 (PMC2729376; doi:10.1371/journal.pone.0006816)

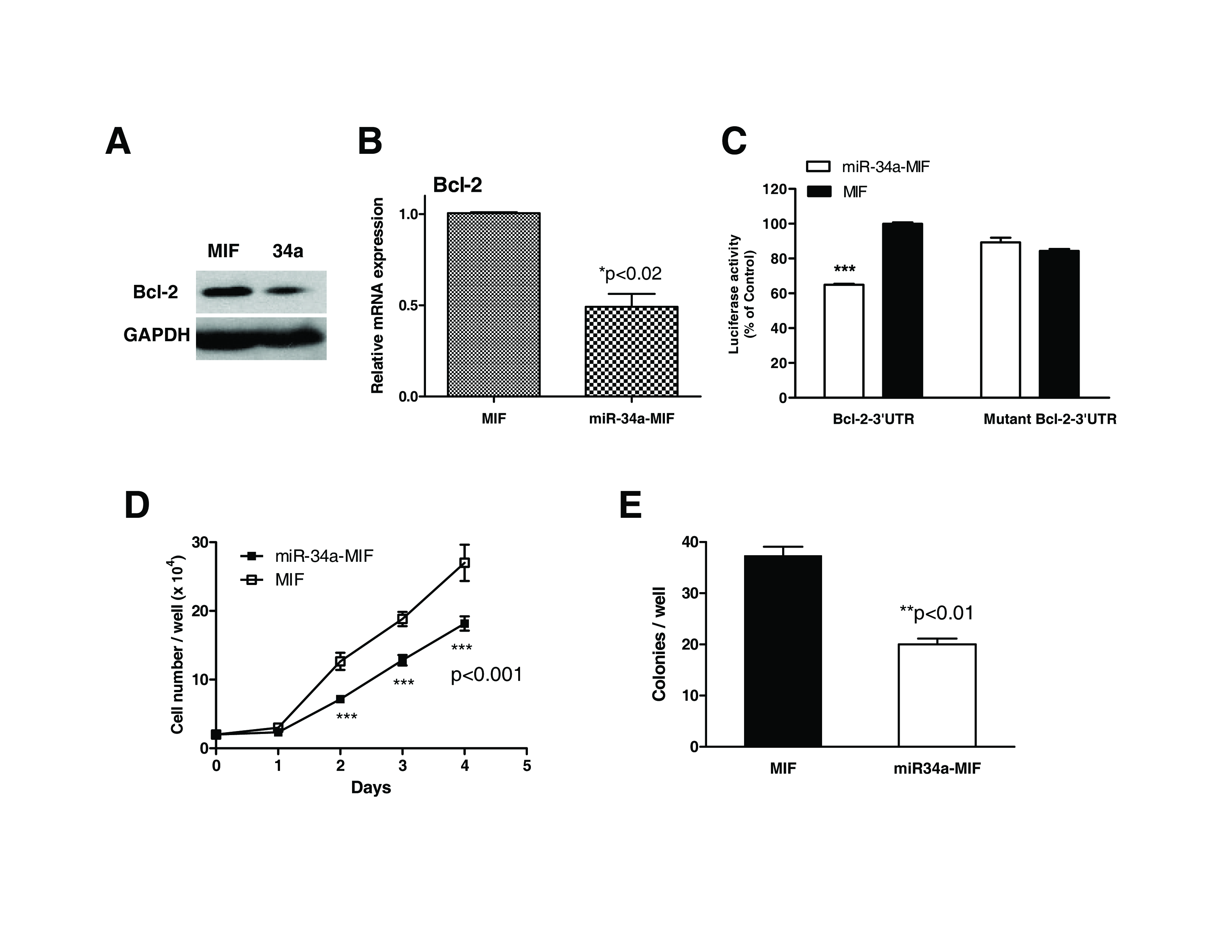

Supplement: Figure S1 — Characterization of the Zeocin-resistant stable MiaPaCa2-miR-34a cells. MiaPaCa2 cells were infected with feline immunodeficiency virus (FIV) lentiviral system expressing miR-34a (miR-34a-MIF) or control (MIF), and selected for stable cells by Zeocin-resistance. A, Western blot shows Bcl-2 protein is downregulated in miR-34a-MIF clone. B, qRT-PCR analysis shows that the target gene Bcl-2 is downregulated in miR-34-MIF clone. C, Bcl-2 3′UTR Luciferase Reporter Assay shows that the miR-34a is functional in miR-34-MIF clone. Error bar indicates S.D. D, miR-34a-MIF cells grows slower than MIF control cells. Cells were plated in a 24-well plate, at every 24 h, cells were harvested in triplicate wells and the viable cells were counted by Trypan Blue exclusion. E, Colony formation assay shows the miR-34a inhibits clonogenic growth of the miR-34-MIF. Cells were seeded in 6-well plate (200 cells/well) in triplicates. After 12–14 days incubation, plates were stained with 0.1% crystal violet. Colonies with over 50 cells were counted. **P<0.01, ***P<0.001, Student's t-test, n = 3. (1.02 MB TIF) [file pone.0006816.s001.tif]

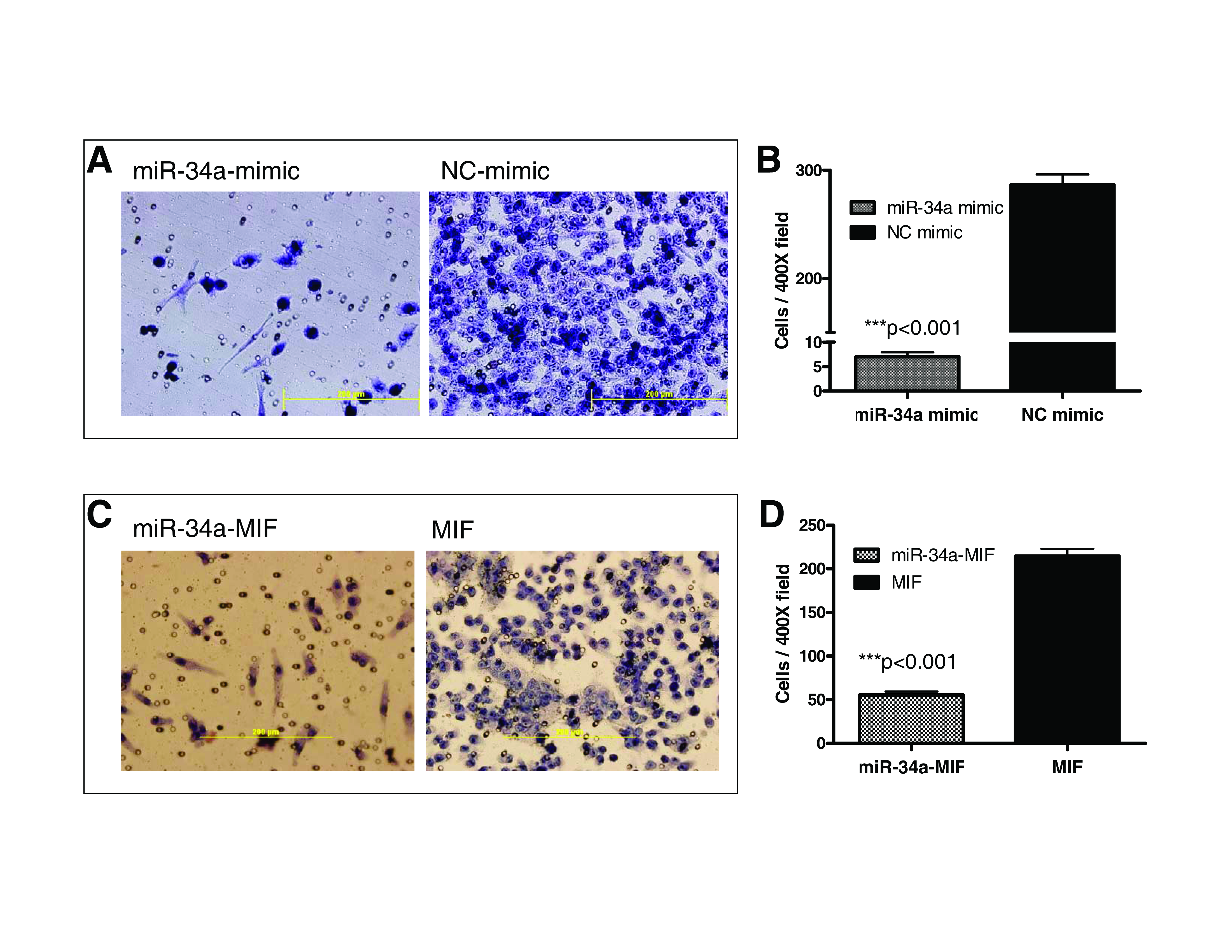

Supplement: Figure S2 — Restoration of miR-34 inhibits the invasion of MiaPaCa2 cells. Cell invasion assay was carried out using Transwells Invasion Kit (Corning Costar) in 24-well tissue culture plates. A–B. MiaPaCa2 cells were transfected with miR-34a mimic or NC mimic and placed in the Transwell inserts, cultured for two days, observed under microscope (A) and quantified (B). C–D. The stable MiaPaCa2-miR-34a-MIF or MiaPaCa2-MIF cells were placed in the Transwell inserts, cultured for two days, observed under microscope (C) and quantified (D). (7.60 MB TIF) [file pone.0006816.s002.tif]

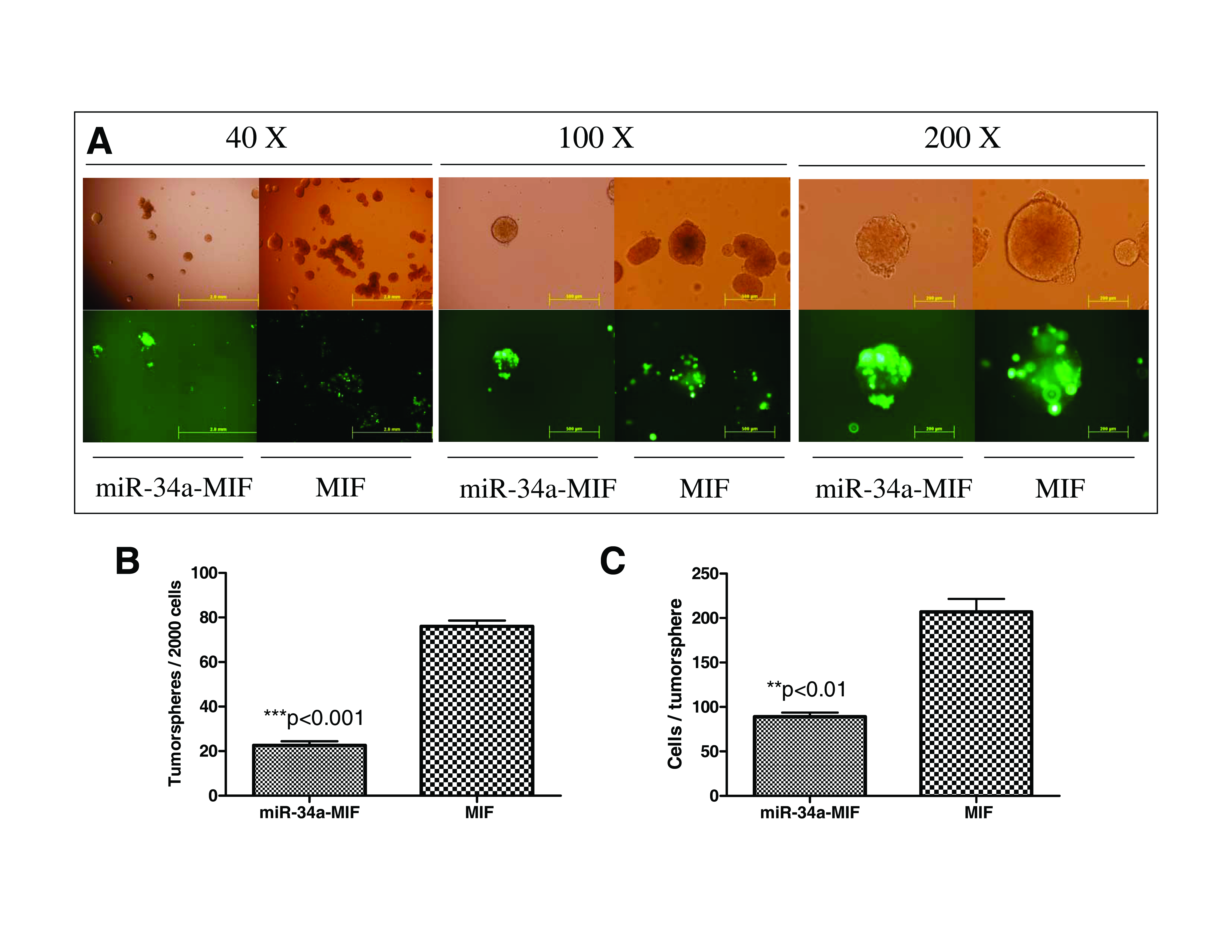

Supplement: Figure S3 — Restoration of miR-34 by MIF lentiviral system inhibited MiaPaCa2 tumorspheres. The stable MiaPaCa2-miR-34a-MIF or MiaPaCa2-MIF cells were plated for tumorsphere formation as described in Materials and Methods. 7–10 days later, tumorspheres were observed under microscope (A) and quantified (B). C. Quantification of cell numbers per tumorsphere. Tumorspheres were collected with a 40 um filter (BD), and dissociated with trypsin for single cell suspension. Cells were counted with trypan blue exclusion and data are presented as number of cells per tumorsphere. **P<0.01, ***P<0.001, Student's t-test, n = 3. (3.07 MB TIF) [file pone.0006816.s003.tif]
